# Narratives in Telehealth (Non-)Adoption Among Korean Older Adult Immigrants: An Exploratory Study

**DOI:** 10.1093/geroni/igaf122.1900

**Published:** 2025-12-31

**Authors:** Sungjae Hong, Ha Young Choi, Shannon Mejia

**Affiliations:** University of Illinois Urbana-Champaign, Champaign, Illinois, United States; University of Illinois Urbana-Champaign, Urbana, Illinois, United States; University of Illinois Urbana-Champaign, Champaign, Illinois, United States

## Abstract

Stay-at-home orders during the COVID-19 crisis accelerated the transition from in-person to digital healthcare. However, older adult immigrant populations remain among the least likely to adopt digital healthcare services. This study explored narratives from older adult immigrants about their experiences with digital healthcare services during the COVID-19 crisis and adoption practices in the years that followed. Our purpose is to reveal hidden components of digital healthcare transition among older adult immigrants in the United States to inform future research on this topic. A total of 22 older Korean adults were recruited from the Chicago metropolitan area during Spring 2024. Semi-structured interviews collected narratives of participants’ digital healthcare service use during and after the COVID-19 crisis. We employed inductive and latent thematic analysis to extract contexts of (not) using digital healthcare. Emergent themes included: 1) selective transition to digital healthcare, 2) phone-based remote healthcare as a potential competitor of digital healthcare, 3) healthcare providers as a key actor determining digital healthcare adoption and use, and 4) healthcare visits as a potential life routine for older adults. This study asks gerontechnologists to consider different modalities of digital health and recognize the roles of diverse stakeholders, including healthcare providers, in shaping digital healthcare adoption and use. Also, this study contributes to digital healthcare inclusivity by shedding light on the nuanced healthcare experiences in later life and health crisis contexts, aspects which might not be fully replaced by digitalized healthcare services.

